# Plasma proteomic profiles of patients with atopic dermatitis with moderate-to-severe pruritus treated with a single dose of nemolizumab

**DOI:** 10.1016/j.xjidi.2026.100457

**Published:** 2026-02-09

**Authors:** Saeko Nakajima, Hajime Iizuka, Kayo Taira, Kentaro Tanaka, Kei Hashimoto, Noriaki Kaneda, Kotaro Iwasaki, Takuya Takafuji, Yoshihito Yamada, Kenji Kabashima

**Affiliations:** 1 Department of Dermatology, Kyoto University Graduate School of Medicine, Kyoto, Japan; 2 Department of Drug Discovery for Inflammatory Skin Diseases, Kyoto University Graduate School of Medicine, Kyoto, Japan; 3 Hosui General Medical Clinic, Research Institute of Psoriasis, Sapporo, Japan; 4 SOUSEIKAI Hakata Clinic, Fukuoka, Japan; 5 Kyoto R&D Center, Maruho, Kyoto, Japan; 6 Medical Affairs Department, Maruho, Osaka, Japan; 7 A∗STAR Skin Research Labs (A∗SRL), Agency for Science, Technology and Research (A∗STAR), Singapore, Singapore; 8 Skin Research Institute of Singapore (SRIS), Agency for Science, Technology and Research (A∗STAR), Singapore, Singapore; 9 Singapore Immunology Network (SIgN), Agency for Science, Technology and Research (A∗STAR), Singapore, Singapore

**Keywords:** Atopic dermatitis, CCL17, IL-31, Nemolizumab, Proteomics

## Abstract

Nemolizumab demonstrates efficacy against pruritus and eczema by inhibiting IL-31 signaling in patients with atopic dermatitis. However, its effect on the systemic immune response at the molecular level remains unknown. In this study, we aimed to elucidate it by investigating plasma proteins and pathways modulated by nemolizumab under concomitant topical treatment. Plasma protein profiling was conducted for 25 Japanese patients with atopic dermatitis who received a single 60 mg dose of nemolizumab at baseline and 1, 2, 4, and 8 weeks after administration using the SomaScan 7k assay. Proteome data were analyzed through differential expression, linear regression, and enrichment analyses, which revealed upregulation of pathways such as neutrophil degranulation and integrin signaling, alongside downregulation of PTEN signaling. Although nemolizumab did not induce substantial changes at the individual protein level, it tended to reverse disease-associated alterations in many key pathways, as revealed by enrichment analysis using proteins with statistically significant but modest expression changes. In addition, we identified pathways and proteins—including TARC (thymus and activation-regulated chemokine)/CCL17—that were associated with pruritus and eczema severity. In conclusion, proteins and pathways involved in the systemic immune response modulated by nemolizumab were identified, potentially reflecting its therapeutic effects. The M525101-05 study (jRCT2080225290) was registered on July 22, 2020, and the MIT-502 study (jRCT1030230474) was registered on November 22, 2023.

## Introduction

Atopic dermatitis (AD) is a prevalent chronic inflammatory skin disorder marked by impaired skin barrier function, type 2 inflammation, and severe pruritus (Guttman-Yassky et al, 2025; Kabashima, 2013; Nakajima et al, 2024). Its pathogenesis is multifactorial, involving a complex interplay of genetic predisposition, immune system dysregulation, barrier dysfunction, and environmental triggers (Nakajima et al, 2024). These interconnected factors contribute to the heterogeneity and complexity of AD. Recent advances in AD treatment, particularly with biologics that target diverse molecular pathways, have improved clinical outcomes and expanded therapeutic options (Bieber, 2022; Guttman-Yassky et al, 2025). Personalized treatment selection based on individual molecular and clinical profile is increasingly recognized as essential for effective AD management. Gene expression analyses using skin biopsies and PBMCs have greatly enhanced our understanding of AD pathophysiology and facilitated the identification of potential biomarkers (Sekita et al, 2023). More recently, serum and plasma protein profiling through proteomics has emerged as a valuable complement to transcriptomic analyses because proteins more accurately reflect biological activity, cellular functions, and dynamic responses than transcripts (He et al, 2022; Wang et al, 2017). Thus, integrating plasma sampling—a minimally invasive approach—with proteomic analysis enables a comprehensive investigation of systemic alterations and facilitates the identification of key biomarkers associated with disease progression and therapeutic response.

IL-31, a critical mediator of pruritus, also plays a role in promoting inflammation and compromising epidermal barrier integrity (Nemmer et al, 2021; Roh et al, 2021). Nemolizumab, a mAb targeting the IL-31 receptor A, blocks IL-31 signaling, thereby interrupting the itch–scratch cycle, reducing eczema severity, and enhancing QOL in patients with moderate-to-severe AD, positioning it as a promising therapeutic option (Kabashima et al, 2022, 2020). However, its systemic molecular effects remain incompletely understood. For example, some patients exhibit a transient increase in TARC (thymus- and activation-regulated chemokine)/CCL17 after treatment, which does not correlate with clinical manifestations such as itch or other skin symptoms. This observation underscores the need for deeper molecular insights into how nemolizumab modulates systemic immune responses.

To investigate the systemic biological effects of nemolizumab, we conducted 2 complementary studies. In the M525101-05 study, Japanese patients with AD received a single 60 mg dose of nemolizumab, and its pharmacological effects were assessed by a ligand-based cytokine assay to monitor changes in multiple cytokines. Subsequently, the MIT-502 study involved the collection of plasma samples from newly recruited healthy controls (HCs). Using these samples, we performed comparative proteomic analyses to characterize systemic changes in the plasma proteome between HCs and patients with AD as well as in patients with AD before and after nemolizumab administration. To elucidate the pathways modulated by nemolizumab, we employed the SomaScan®7k assay—an advanced proteomic platform that simultaneously quantifies approximately 7000 plasma proteins with high specificity and affinity using SOMAmers (Slow Off-rate Modified Aptamers) (Kraemer et al, 2024). This approach provides deeper insights into the molecular mechanisms of nemolizumab and its potential clinical benefits.

## Results

### Baseline characteristics of patients with AD

**Figure 1:**
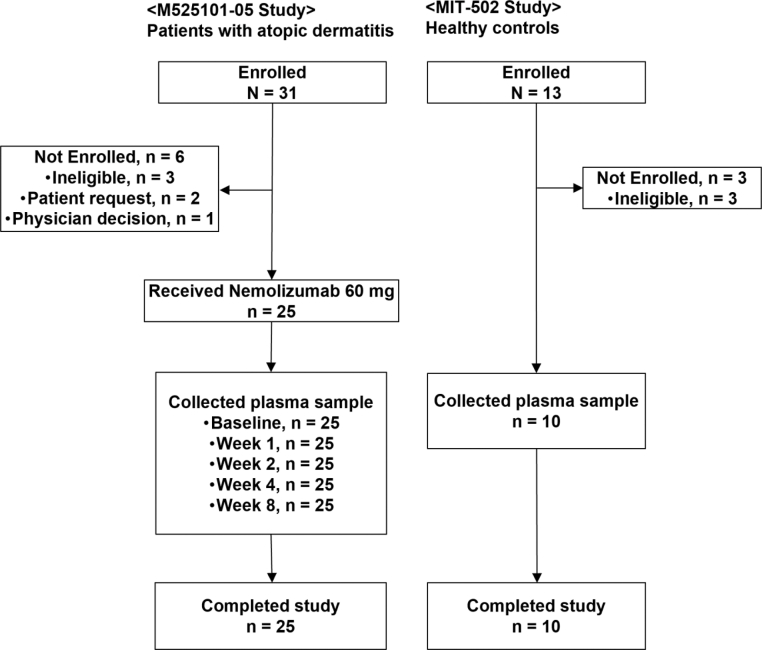
**Overview of study design and sample collection timeline in 2 complementary cohorts.** In the M525101-05 study, 25 of 31 patients with AD who provided informed consent were enrolled and completed the trial. Each participant received a single 60 mg dose of nemolizumab and underwent plasma sampling at baseline and 1, 2, 4, and 8 weeks after administration. Six patients were excluded owing to ineligibility (n = 3), withdrawal by patient request (n = 2), or physician decision (n = 1). In the MIT-502 study, 10 of 13 HCs who consented were enrolled and completed plasma collection at a single time point. Three individuals were excluded owing to ineligibility. AD, atopic dermatitis; HC, healthy control.

Twenty-five of 31 enrolled patients with AD received a single dose of 60 mg nemolizumab with topical treatment and underwent plasma collection at baseline and 1, 2, 4, and 8 weeks after the administration (Figure 1). Plasma samples were also collected from 10 HCs. The demographic and baseline characteristics, including age, sex, body height, and weight, were comparable between patients with AD and HCs (Table 1). In patients with AD, baseline scores (mean ± SD) of Peak Pruritus Numeric Rating Scale (PP-NRS), the itch score, and Eczema Area and Severity Index (EASI) were 7.4 ± 1.7, 3.2 ± 0.4, and 27.97 ± 9.97, respectively. The baseline plasma concentrations (mean ± SD) of TARC and IgE were 3125.2 ± 3785.8 ng/l (normal range <450 ng/l) and 20,266.6 ± 15,691.3 μg/l (normal range ≤408 mg/l), respectively (Table 1).

**Table 1:** Demographic and Baseline Clinical Characteristics in M525101-05 and MIT-502 Studies

Characteristics	AD M525101-05 Study n = 25	HC MIT-502 Study n = 10
Age, y, mean (SD)	35.2 (11.8)	31.2 (3.6)
Sex (male), n (%)	19 (76.0%)	7 (70.0%)
Height (cm), mean (SD)	167.09 (6.83)	170.50 (7.23)
Weight (kg), mean (SD)	63.01 (12.29)	60.00 (10.10)
BMI (kg/m^2^), mean (SD)	22.41 (3.21)	20.51 (2.32)
Allergic disease	12 (48.0%)	—
Asthma bronchiale, n (%)	6 (24.0%)	
Pollinosis, n (%)	4 (16.0%)	
Allergic rhinitis, n (%)	6 (24.0%)	
Allergic conjunctivitis, n (%)	2 (8.0%)	
Food allergy, n (%)	5 (20.0%)	
Duration of primary disease, y, mean (SD)	26.66 (13.15)	—
PP-NRS, mean (SD)	7.4 (1.7)	—
Itch score, mean (SD)	3.2 (0.4)	—
EASI, mean (SD)	27.97 (9.97)	—
BSA, mean (SD)	47.12 (19.09)	—
TARC (ng/l), mean (SD)	3125.2 (3785.8)	—
IgE (μg/l), mean (SD)	20,266.6 (15,691.3)	—

Abbreviations: AD, atopic dermatitis; BMI, body mass index; BSA, body surface area; EASI, Eczema Area and Severity Index; HC, healthy control; PP-NRS, Peak Pruritus Numeric Rating Scale.

### Efficacy and safety of nemolizumab in M525101-05 study

**Figure 2:**
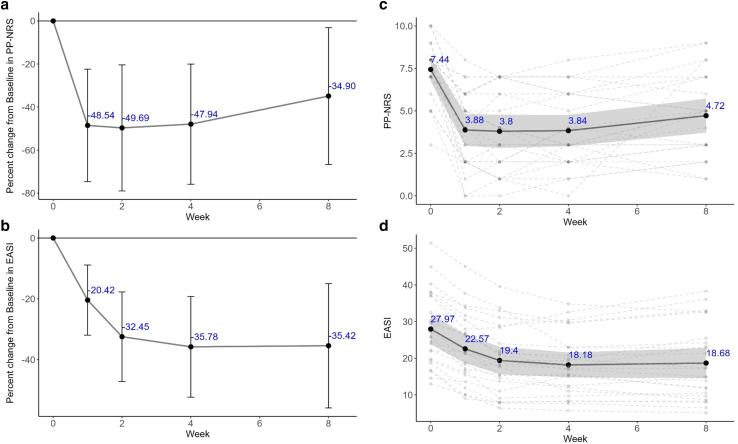
**Time course of pruritus and eczema severity scores after a single dose of nemolizumab in patients with AD.** (**a, b**) Percentage change from baseline (mean ± SD) in PP-NRS and EASI scores after a single dose of nemolizumab. No formal statistical tests were performed. (**c, d**) Individual patient scores over time for PP-NRS and EASI, with mean values and 95% CIs. Solid lines represent group means (n = 25), with error bars or shaded areas indicating SD or 95% CIs, respectively. Dashed lines indicate longitudinal changes for individual patients. AD, atopic dermatitis; CI, confidence interval; EASI, Eczema Area and Severity Index; PP-NRS, Peak Pruritus Numeric Rating Scale.

As shown in Figure 2, patients with AD treated with a single dose of 60 mg nemolizumab exhibited substantial reductions in both PP-NRS and EASI, reflecting improvements in pruritus and eczema severity. The mean percentage change in PP-NRS from baseline was −48.5% at week 1, sustained through week 4 (−47.9%), and slightly attenuated by week 8 (−34.9%). Similarly, EASI scores decreased by −20.4% at week 1 and reached approximately −35% from week 4 onward. Individual patient trajectories, along with group means and 95% confidence intervals, are illustrated in Figure 2c and d.

Regarding safety, adverse events were reported in 5 of the 25 enrolled patients, with only 1 case deemed related to the study drug. No severe adverse events occurred; 2 were classified as moderate, and 4 were classified as mild. Additional adverse events of clinical interest—such as cellulitis and Kaposi's varicelliform eruption—were reported in 4 patients but were not considered treatment related (Table 2).

**Table 2:** TEAEs in M525101-05 Study

Adverse Event	Nemolizumab 60 mg, Single Dose (n = 25) n (%)
Any TEAE	5 (20.0)
Severe	0
Moderate	2 (8.0)
Mild	4 (16.0)
Serious TEAEs	0
Death	0
Treatment modifications (including discontinuation, interruption, and dose reduction)	0
Other significant TEAEs	
Cellulitis	1 (4.0)
Skin infection	1 (4.0)^1^
Kaposi's varicelliform eruption	1 (4.0)^1^
Asthma	1 (4.0)^1^

Abbreviation: TEAE, treatment-emergent adverse event.

1 None were considered to be treatment related by the investigator.

### Baseline proteomic profile of patients with AD

**Figure 3:**
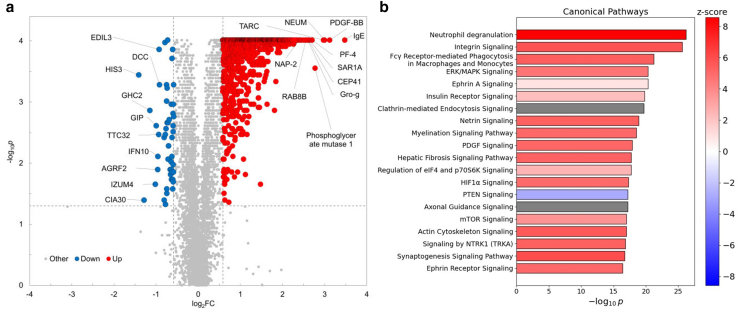
**Baseline proteomic profile of patients with AD.** (**a**) Volcano plot of DEPs comparing plasma samples from patients with AD at baseline with those of HCs. The x-axis shows the log_2_ FC, and the y-axis shows the −log_10_*P*-value. *P*-values were adjusted for multiple testing using the BH method. Dashed lines indicate the thresholds for defining DEPs: log_2_FC ≥ 0.58 and FDR < 0.05. Proteins upregulated in AD are shown in red, those downregulated are in blue, and nonsignificant proteins are in gray. (**b**) Bar plot showing top-ranked canonical pathways identified by IPA using DEPs. Bar colors indicate predicted pathway activity: red for positive Z-scores (activation), blue for negative Z-scores (inhibition), and gray for pathways where the Z-score was not calculable. AD, atopic dermatitis; BH, Benjamini–Hochberg; DEP, differentially expressed protein; FC, fold change; FDR, false discovery rate; HC, healthy control; IPA, Ingenuity Pathway Analysis.

To explore the systemic proteomic alterations associated with AD, we first compared plasma protein profiles between patients with AD and HCs using differential expression analysis. The plasma proteomic analysis identified significant differences in multiple differentially expressed proteins (DEPs) in the baseline samples between patients with AD and HCs (Figure 3a). A total of 1181 proteins were significantly upregulated, and 49 proteins were significantly downregulated in patients with AD compared with those in HCs, as detected by the SomaScan assay (log_2_ fold change [FC] ≥ 0.58, false discovery rate [FDR] < 0.05). T helper 2–associated molecules, including IgE and TARC/CCL17, platelet factor 4, neutrophil-related proteins (neutrophil-activating peptide 2 and phosphoglycerate mutase 1), and neuromodulin (NEUM)/growth-associated protein-43 were significantly elevated in patients with AD. Among these, IgE—an established biomarker of disease activity in AD along with TARC/CCL17 (Wang et al, 2017)—was the most significantly elevated. Enrichment analysis using DEPs from the baseline samples between patients with AD and HC group identified several immune-related biological processes in patients with AD. Neutrophil degranulation, integrin signaling, extracellular signal–regulated kinase/MAPK signaling, and mTOR (mammalian target of rapamycin) signaling were upregulated, whereas PTEN (phosphatase and tensin homolog) signaling was downregulated (Figure 3b). These findings confirm that patients with AD exhibit a systemic proteomic signature characterized by T helper 2–skewed inflammation, neutrophil activation, and dysregulation of key signaling pathways such as mTOR and PTEN.

### Temporal proteomic changes after nemolizumab administration

**Figure 4:**
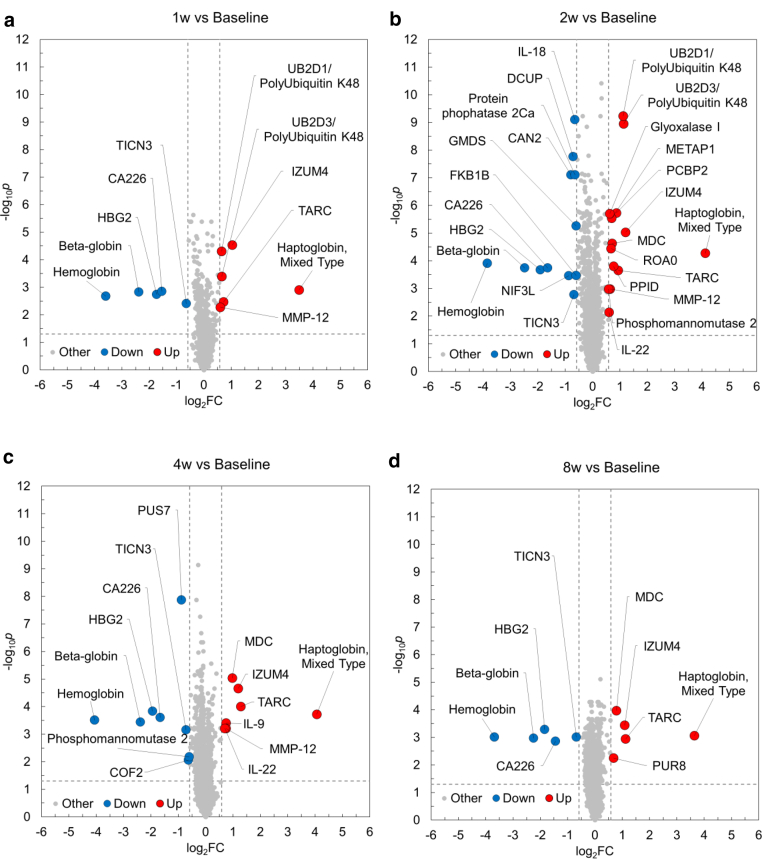
**Proteomic changes in patients with AD after nemolizumab administration.** (**a–d**) Volcano plots showing DEA of plasma proteins 1, 2, 4, and 8 weeks after nemolizumab administration, compared with baseline. The x-axis shows log_2_FC, and the y-axis shows −log_10_*P*-values, adjusted for multiple testing using the BH method. Dashed lines indicate the thresholds for defining DEPs: FDR < 0.05 and log_2_FC ≥ 0.58. Upregulated DEPs are shown in red, downregulated ones are in blue, and nonsignificant proteins are in gray. (**e, f**) Heatmaps displaying Z-scores for canonical pathways identified by IPA. Panel **e** shows the top 20 and bottom 20 pathways. The pathways are selected and ranked by the Z-score from the baseline comparison between HCs and patients with AD (ie, pathways most significantly affected in patients with AD at baseline). Panel **f** shows the pathways derived from the same pathway analysis. However, the pathways are selected and ranked by the maximum absolute Z-score observed for each pathway across the 4 post-treatment comparisons (ie, pathways most significantly affected by nemolizumab treatment). AD, atopic dermatitis; BH, Benjamini–Hochberg; DEA, differential expression analysis; FC, fold change; FDR, false discovery rate; HC, healthy control; IPA, Ingenuity Pathway Analysis.

**Figure 5:**
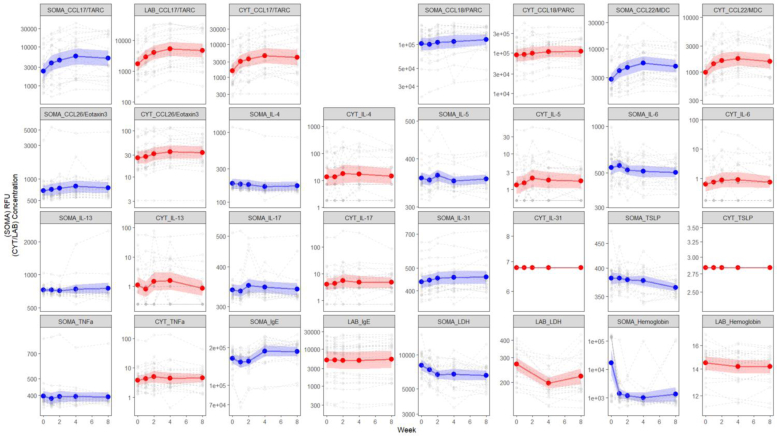
**Changes in cytokines and clinical laboratory parameters assessed by SomaScan assay (SOMA) and conventional methods (CYT and LAB).** Blue lines represent SOMA data, and redlines represent multiplex cytokine assays (denoted as CYT) or clinical laboratory tests (denoted as LAB) data. Solid lines indicate mean values (n = 25), with shaded areas representing 95% CIs. Dashed lines represent individual subject longitudinal changes. CYT: multiplex cytokine assays; LAB: clinical tests; Y-axis units are SOMA (RFU), CYT (pg/ml), and LAB (CCL17/TARC: ng/l; IgE: μg/l; LDH: U/l; hemoglobin: g/l). CI, confidence interval; LDH, lactate dehydrogenase; RFU, relative fluorescent unit.

Next, to evaluate the effects of nemolizumab treatment on the plasma proteome, we compared protein profiles at multiple time points (baseline and 1, 2, 4, and 8 weeks) after a single dose of nemolizumab. Although many proteins exhibited statistically significant changes (FDR < 0.05), the corresponding log_2_FCs were modest in magnitude, and only a limited subset met the predefined FC threshold (log_2_FC ≥ 0.58) (Figure 4a–d). At all or multiple time points after nemolizumab administration, TARC/CCL17, macrophage-derived chemokine (MDC)/CCL22, polyubiquitin K48, IZUM4 (izumo sperm-egg fusion protein 4), and matrix metalloproteinase 12 were increased, whereas testican 3 was decreased. Notably, haptoglobin levels markedly increased, and hemoglobin and hemoglobin subunit β/γ2 levels decreased at all post-treatment time points. The changes in cytokine and laboratory values after a single dose of nemolizumab were compared between the measurements obtained from SomaScan and those from conventional assays. The results showed that fluctuations in plasma proteins measured by both methods exhibited similar patterns. Although few consistent changes were observed overall, chemokines such as TARC/CCL17 and MDC/CCL22 tended to increase, which contrasts with the observed clinical improvement, and lactate dehydrogenase levels tended to decrease (Figure 5). Some proteins were uniquely detected by the SomaScan assay or showed divergent trends between the SomaScan and conventional assay. The discrepancy is likely attributable to differences in detection mechanisms. Although the FCs of individual proteins were modest, nemolizumab administration resulted in consistent temporal shifts in several immunologically relevant proteins, including chemokines and hemoglobin-related markers.

### Nemolizumab treatment mitigates AD-related pathway dysregulation toward a healthy state

The number of DEPs between baseline and each time point peaked at 2 weeks, with a total of 26 DEPs (14 upregulated and 12 downregulated). Because relying solely on DEPs was deemed insufficient for enrichment analysis, we performed the analysis using proteins with FDR < 0.05, irrespective of the log_2_FC threshold. Z-scores were used to compare both the direction and magnitude of pathway changes observed in patients with AD (baseline AD vs HC) and those after nemolizumab administration (1, 2, 4, and 8 weeks vs baseline). Most pathways (246 in total) that showed large deviations at baseline in patients with AD demonstrated a tendency toward normalization after nemolizumab treatment, with this trend sustained through 8 weeks (Figure 4e). Many pathways that were downregulated after nemolizumab treatment had positive Z-scores at baseline in patients with AD. The minimum Z-scores observed up to 8 weeks for ubiquitination-related pathways (deubiquitination, protein ubiquitination pathway), cell division–related pathways (cell cycle checkpoints, mitotic metaphase and anaphase), and the neutrophil extracellular trap signaling pathway were adjusted to around −5.0. Conversely, several pathways (14 in total) that were upregulated at baseline in patients with AD such as extracellular matrix organization were further elevated after treatment, although the maximum Z-score remained below 2.7 (Figure 4f). Pathway-level analysis revealed an overall trend toward normalization of AD-associated dysregulation after nemolizumab treatment, suggesting systemic immunomodulatory effects beyond itch control.

### Association between plasma proteins and clinical outcomes

**Figure 6:**
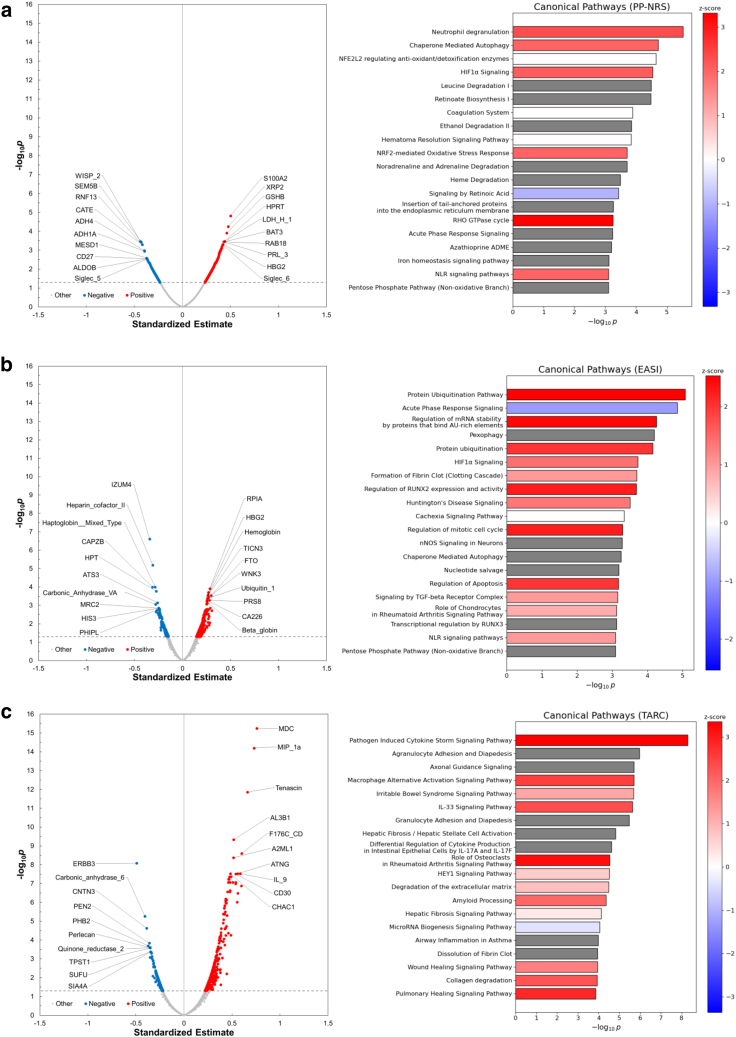
**Plasma proteins associated with PP-NRS, EASI, and serum TARC/CCL17 in patients with AD.** (**a–c)** The volcano plots from regression analysis, which identified plasma proteins significantly associated with (**a**) PP-NRS, (**b**) EASI, and (**c**) serum TARC/CCL17 levels in patients with AD, show the standardized beta estimates on the x-axis and the negative log_10_*p*-values, adjusted for multiple testing using the BH method, on the y-axis. Red dots: standardized beta estimate > 0 and FDR < 0.05; blue dots: standardized beta estimate < 0 and FDR < 0.05; gray dots: not significance. (**a–c)** Right: The bar plots displaying the top-ranked canonical pathways identified using IPA. Red: Z score > 0; blue: Z score < 0; white: Z score = 0; gray: Z score not calculable. AD, atopic dermatitis; BH, Benjamini–Hochberg; EASI, Eczema Area and Severity Index; FDR, false discovery rate; IPA, Ingenuity Pathway Analysis; PP-NRS, Peak Pruritus Numeric Rating Scale.

To identify protein- and pathway-level correlates of clinical outcomes such as pruritus and eczema severity, we conducted linear regression analyses between proteomic data and PP-NRS, EASI, and TARC/CCL17 levels. For this analysis, proteomics data collected at all time points (baseline and at 1, 2, 4, and 8 weeks after administration) from 25 patients with AD were pooled and integrated. Linear regression analysis identified 1038 proteins correlated with PP-NRS (851 positively and 187 negatively) (Figure 6a), 945 with EASI (765 positively and 180 negatively) (Figure 6b), and 547 with TARC/CCL17 (353 positively and 194 negatively) (Figure 6c). Ingenuity Pathway Analysis (IPA) of the top 200 proteins revealed that PP-NRS was positively associated with neutrophil degranulation, chaperone-mediated autophagy pathways, and RHO GTPase cycle (Figure 6a). EASI was positively linked to ubiquitination-related pathways, including protein ubiquitination pathway and protein ubiquitination (Figure 6b). TARC/CCL17 levels were positively associated with MDC and MIP-1α proteins. IPA further showed that pathogen-induced cytokine storm signaling pathway and macrophage alternative activation signaling pathway were positively correlated with TARC/CCL17 levels (Figure 6c). The observed correlations between clinical indices and specific proteomic changes highlight potential mechanistic links and candidate biomarkers relevant to treatment response and disease activity.

## Discussion

This study provides, to our knowledge, a previously unreported comprehensive plasma proteomic analysis of patients with AD before and after a single dose of nemolizumab. In our cohort, a single 60 mg dose of nemolizumab, administered alongside medium-potency topical glucocorticoids, resulted in significant improvements in pruritus and eczema severity. Reductions in PP-NRS and EASI were observed as early as 1 week after administration and were sustained through week 8. These results are in line with previous reports despite methodological differences in pruritus assessment (Kabashima et al, 2020). Moreover, nemolizumab was well-tolerated, with no severe events and no exacerbations of AD during observation period. Although changes at the individual protein level were modest, as expected after a single administration, pathway-level analysis revealed systemic immunological alterations associated with clinical improvement. These findings indicate that nemolizumab may influence systemic immune and inflammatory signaling pathways beyond its primary role in pruritus suppression.

At baseline, plasma proteomic profiles of patients with AD exhibited characteristic immune alterations compared with those of HCs. Notably, T helper 2–associated molecules such as TARC/CCL17 and IgE were significantly elevated, along with proteins associated with neutrophil activation. Although hemoglobin levels remained stable in clinical laboratory values, an apparent reduction was seen in the SomaScan data (Figure 5). This discrepancy likely reflects hemolysis in some plasma samples at baseline and is not considered clinically meaningful, as also supported by a concurrent decrease in haptoglobin levels. Enrichment analysis of DEPs revealed activation of multiple immune-related pathways, including neutrophil degranulation, integrin signaling (Sonnenberg-Riethmacher et al, 2021), extracellular signal–regulated kinase/MAPK signaling (Kabashima and Irie, 2021; Zeze et al, 2022), and mTOR signaling (Gupta et al, 2023; Roy et al, 2023). In contrast, PTEN signaling, a known negative regulator of mTOR (Roy et al, 2023), was downregulated. These findings align with those of earlier transcriptomic and proteomic studies in both Japanese and Western AD cohorts (Sekita et al, 2023; Wang et al, 2017), reinforcing the systemic nature of immune dysregulation in AD and underscoring the relevance of these pathways as potential therapeutic targets or biomarkers.

After nemolizumab treatment, pathway-level modulation was evident despite the small FCs in individual protein abundance. In adolescents with moderate-to-severe AD treated with nemolizumab, several protein levels in stratum corneum were associated with clinical improvement, whereas plasma protein levels showed no association with clinical scores (Sidbury et al, 2022). Consistent with this finding, the effect of nemolizumab administration on systemic proteomics appeared to be minimal in our study. The majority of dysregulated pathways in patients with AD showed partial or full reversal toward the healthy state, as indicated by enrichment analysis on the basis of proteins with significant but subtle expression changes. This normalization trend was particularly notable in ubiquitination-, cell division–, and neutrophil-related pathways. In addition, extracellular matrix–related proteins such as matrix metalloproteinase 12 remained elevated after improvement in skin inflammation, likely reflecting reparative remodeling and ongoing tissue repair (Potekaev et al, 2021). Our enrichment analysis using IPA indicated that deubiquitination, protein ubiquitination pathway, and cell cycle checkpoints as the top pathways, and synaptogenesis signaling pathway was also downregulated as a neuron-related pathway after nemolizumab treatment. These findings are partially consistent with prior proteomic studies conducted in patients with prurigo nodularis treated with nemolizumab, in which similar pathways were identified (Deng et al, 2023; Tsoi et al, 2022). On the basis of these concordant results, we interpret these pathway changes as downstream consequences of IL-31 signaling inhibition. Interestingly, some chemokines such as TARC/CCL17 and MDC/CCL22 remained elevated or even increased after treatment, despite clear clinical improvement. In the previous study of patients with AD, serum TARC levels decreased beginning at 4 weeks after dupilumab initiation, whereas TARC remained elevated despite clinical improvement during treatment with Jak inhibitors (Boesjes et al, 2024). These apparent dissociations between chemokine levels and symptom severity suggest that these molecules may reflect various immune remodeling processes rather than direct disease activity. For example, increased expression of TARC/CCL17 and MDC/CCL22 may be related to the expansion or activation of regulatory dendritic cells, which possess both proinflammatory and immunosuppressive functions, suggesting that their increase after nemolizumab treatment could drive chemokine production while simultaneously exerting inhibitory effects on inflammation (Li et al, 2023).

**Figure 7:**
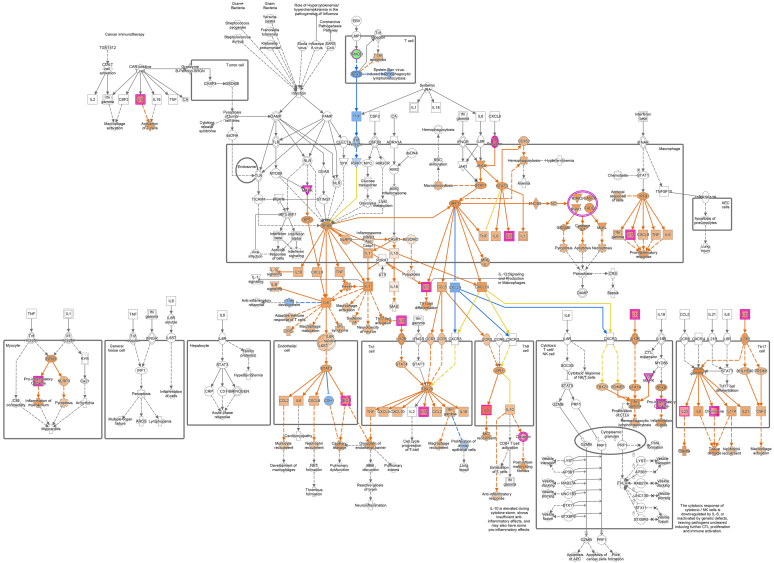
**Overlapping of “pathogen-induced cytokine storm” pathway and TARC/CCL17-related proteins.** The figure shows the “pathogen-induced cytokine storm” pathway overlapping with several proteins significantly related to TARC/CCL17 (BH-adjusted *P* < .05 and the absolute beta estimate ranked within the top 200), colored by standardized beta estimate value and predicted relationships. Red: standardized beta estimate > 0; green: standardized beta estimate < 0; orange: predicted relationships leading to activation; blue: predicted relationships leading to inhibition; yellow: predicted relationships representing findings inconsistent with the state of the downstream molecule; gray: relationships not predicted; dashed lines: indirect relationship; solid lines: direct relationship. BH, Benjamini–Hochberg.

To further investigate the relationship between molecular changes and clinical response, we performed regression analyses correlating proteomic data with PP-NRS and EASI. Pruritus severity was positively associated with several immune-related pathways, including neutrophil degranulation, chaperone-mediated autophagy, and Rho GTPase signaling—pathways known to contribute to inflammatory signaling and sensory neuron plasticity (Kalpachidou et al, 2019; Yao and Shen, 2023). Conversely, retinoic acid signaling, which has been shown to inhibit autophagy, exhibited a negative association with pruritus scores (Anguiano et al, 2013). Eczema severity was linked to pathways involved in protein ubiquitination, cell cycle progression, and epithelial proliferation—represented by regulation of mitotic cell cycle and regulation of apoptosis, which may underlie skin thickening and barrier impairment in AD (Acevedo et al, 2020; Ma et al, 2022; Szymański et al, 2021; Zhu et al, 2022). In addition, elevated TARC/CCL17 levels were associated with MDC/CCL22, MIP-1α/CCL3, and IL-9 as well as with T helper 17–, IL-12–, T helper 9– (especially in pathogen-induced cytokine storm signaling pathway) (Figure 7), and macrophage-related pathways, indicating various immune network engagement during nemolizumab treatment. Although these analyses are exploratory and based on a limited sample size, they provide a first-systems-level view of how the antipruritic efficacy of IL-31 receptor blockade is reflected in systemic pathway modulation.

This study has several limitations. This study did not include a placebo-treated group as a control. The sample size was relatively small, and patients, who were exclusively Japanese, were allowed to continue various topical agents, introducing potential variability. Furthermore, only a single dose of nemolizumab was administered, limiting our ability to assess cumulative or long-term effects. The post-treatment pathway analysis was based on proteins with FDR <0.05, without a stringent FC threshold, which could include subtle or biologically ambiguous changes. Direct comparisons with other biologics were not feasible owing to unavailability of datasets; however, such comparative analyses across different therapies would warrant investigation in future studies. Despite these limitations, our findings provide insights into the systemic immunomodulatory effects of nemolizumab and identify candidate pathways and protein markers that may be associated with treatment response.

In conclusion, we analyzed the plasma proteomics of Japanese patients with AD using the SomaScan assay and found that pathways such as neutrophil degranulation and integrin signaling were upregulated, whereas PTEN signaling was downregulated. A single dose of nemolizumab did not produce notable changes in the plasma proteome but tended to reverse alterations in nearly all key pathways characteristic of AD, as indicated by enrichment analysis based on proteins with small expression changes. These findings may reflect the therapeutic effect of nemolizumab.

## Materials and Methods

### Study design and patient population

We conducted 2 studies (M525101-05 and MIT-502) in accordance with the ethical principles of the Declaration of Helsinki, Good Clinical Practice guidelines, and all other applicable regulatory requirements. Study documentation was approved by institutional review boards. These studies were registered with the Japan Registry of Clinical Trials under the identifiers jRCT2080225290 (M525101-05) and jRCT1030230474 (MIT-502). Written informed consent was obtained from patients with AD and HCs prior to study initiation. Eligible patients with AD (n = 25) were aged 13–60 years, had a body weight between 30.0 and 120.0 kg, and had a confirmed diagnosis of AD (according to the Hanifin and Rajka criteria [Hanifin and Rajka, 1980]) accompanied by pruritus. At the time of informed consent, patients were required to have a score ≥3 on a 5-level itch scale and a score ≥10 on the EASI, indicating an inadequate pruritic response despite treatment with medium- or high-potency topical corticosteroids or topical calcineurin inhibitors administered at a stable dose for ≥4 weeks and oral antihistamines at a stable dose for ≥2 weeks. Exclusion criteria included any clinically significant medical condition that could pose a risk to the patient or render them unsuitable for study participation, abnormal liver enzyme or hematologic laboratory values, or the presence of conditions likely to interfere with the evaluation of eczema and pruritus. Patients with AD received a single 60 mg dose of nemolizumab under concomitant stable use of medium-potency topical glucocorticoids and were evaluated for efficacy using PP-NRS, EASI, and the itch score at baseline and 1, 2, 4, and 8 weeks after administration. No formal statistical tests were performed for these exploratory efficacy endpoints. Safety endpoints included treatment-emergent adverse events, with severity classified by investigators as mild (discomfort not limiting daily activities), moderate (discomfort affecting daily activities), or severe (interfering with work or normal daily activities). HC participants (n = 10) were aged 20–60 years, weighed between 40.0 and 100.0 kg, and had no history or comorbidities related to AD or other allergic diseases.

### Cytokine assay in M525101-05 study

The Bioplex 200 platform was utilized to quantify multiple target proteins in plasma samples, which were collected in EDTA-2K tubes and stored at −60 °C or below until analysis. Luminex bead–based immunoassays were conducted for TARC/CCL17, PARC (pulmonary and activation-regulated CC chemokine)/CCL18, MDC/CCL22, IL-4, IL-5, IL-6, IL-13, IL-17, IL-31, TNF-α, and TSLP (thymic stromal lymphopoietin), following the manufacturer’s instructions. The concentration of eotaxin-3/CCL26 was determined using a specific ELISA kit.

### SomaScan 7k assay

Plasma samples from 25 patients with AD and 10 HCs were stored at ≤ −60 °C (patients with AD: up to 37 months without freeze–thaw cycles; HCs: approximately 2 months) and analyzed using the SomaScan 7k platform (SomaLogic, Boulder, CO, version 4.1) at FonesLife (Tokyo, Japan). Plasma was incubated with SOMAmer reagents, and protein–SOMAmer complexes were captured using streptavidin-coated magnetic beads. After washes to eliminate nonspecific binding, each bound SOMAmer was eluted and hybridized to custom Agilent DNA microarrays. Fluorescent signals were detected using the Agilent SureScan Microarray Scanner and expressed as relative fluorescent units (RFUs). Normalization was performed in 3 steps: (i) hybridization normalization using internal controls, (ii) median normalization within each 96-well plate, and (iii) calibration normalization using plate-scale factors (Kraemer et al, 2024). The plasma proteomic data from the SomaScan assay were curated to ensure suitability for analysis: only human proteins were retained, and RFU values for each sample were screened for outliers. Plasma proteomic factors with adequate signal-to-noise ratios were selected. Differential expression analysis, detailed in the following section, was then conducted to identify factors exhibiting significantly elevated RFU values in the assay samples.

### Differential expression analysis

The RFU values for each sample from the HC and patient groups were initially subjected to log_2_ transformation. Subsequently, statistical tests were independently performed for each SOMAmer. For comparisons within the 25 patients with AD—between baseline and subsequent time points—a paired-samples *t*-test was used, whereas the Mann–Whitney *U* test was applied to compare baseline samples from 25 patients with AD with those from 10 HCs. The resulting *P*-values were adjusted using the Benjamini–Hochberg method to control the FDR. Log_2_FC was calculated to quantify the average change in protein expression between case and control samples. SOMAmer targets meeting the thresholds of log_2_FC ≥0.58 and FDR <0.05 were classified as DEPs (Berrone et al, 2023; He et al, 2020; Koch et al, 2023; Wang et al, 2017). All statistical analyses were performed using the Python package SciPy (1.12.0) (Virtanen et al, 2020).

### Linear regression analysis

Regression analysis was conducted to identify proteins associated with EASI and PP-NRS—clinical indicators of treatment efficacy—and TARC/CCL17, a marker of disease activity. For this analysis, proteomics data collected at all time points (baseline and at 1, 2, 4, and 8 weeks after administration) from 25 patients with AD were pooled and integrated with corresponding clinical test values for EASI, PP-NRS, and TARC/CCL17, resulting in 125 data points. All variables were normalized using Z-score transformation. Linear regression models were then constructed for each SOMAmer, with EASI, PP-NRS, or TARC/CCL17 as dependent variables and the log_2_ RFU values of each SOMAmer as the explanatory variable. In the EASI model, body surface area and the number of rashes—both strongly correlated with EASI—were included as covariates. No patient background variables showed high correlation with PP-NRS. The number of rashes, which was highly correlated with TARC/CCL17, was included as a covariate in the TARC/CCL17 model. After model construction, analysis of variance was performed, and *P*-values were adjusted for multiple testing using the Benjamini–Hochberg method. These regression analyses were conducted using the R package glmnet (4.1.8) (Tay et al, 2023).

### Enrichment analysis

The enrichment analysis was performed to ensure a consistent interpretation of the differential expression analysis results. The expression changes of DEPs were considered as the expression changes of each gene constituting each DEP. In cases where the same gene appeared in different DEPs, only those showing the same direction of change were retained, and among them, only the result with the lowest *P*-value after Benjamini–Hochberg correction was selected. If the Benjamini–Hochberg–corrected *P*-values were identical, the result with the larger absolute value of log_2_FC was chosen. The following protein sets were subjected to analysis using IPA (Qiagen) (Krämer et al, 2014): (i) DEPs identified in the baseline AD versus HC comparison (log_2_FC ≥0.58 and FDR <0.05); (ii) significant proteins at 1, 2, 4, and 8 weeks compared with those at baseline (FDR <0.05); and (iii) significant top-ranked proteins related to clinical factors on the basis of linear regression analysis (FDR < 0.05 and absolute estimates within the top 200). The canonical pathways associated with these proteins were identified using the “Core Analysis” function of IPA. In this analysis, a *P*-value (right-tailed Fisher exact test) was used to quantify the overlap, and a Z-score was calculated to determine the likelihood and direction (up or downregulated) of pathway activity.

## Ethics Statement

These studies (M525101-05 and MIT-502) were conducted in accordance with the ethical principles of the Declaration of Helsinki, Good Clinical Practice guidelines, and all other applicable regulatory requirements. Study documentations were approved by the institutional review boards (Hakata clinical institutional review board, Institutional Review Board of Hosui General Medical Clinic for M525101-05, and Japan Conference of Clinical Research for MIT-502). All patients and healthy volunteers provided written informed consent.

## Data Availability Statement

Data will be made available on reasonable request from Maruho at info_mrh.ma01@mii.maruho.co.jp.

## ORCIDs

Saeko Nakajima: https://orcid.org/0000-0003-0831-1447

Kentaro Tanaka: http://orcid.org/0009-0001-0844-9369

Kei Hashimoto: http://orcid.org/0000-0002-9089-1227

Noriaki Kaneda: http://orcid.org/0009-0008-4265-8827

Kotaro Iwasaki: http://orcid.org/0009-0005-3090-2851

Takuya Takafuji: http://orcid.org/0009-0001-8643-0014

Yoshihito Yamada: http://orcid.org/0000-0003-4370-7594

Kenji Kabashima: http://orcid.org/0000-0002-0773-0554

## Conflict of Interest

SN has received honoraria as a speaker/consultant for Sanofi, Regeneron, Eli Lilly, Abbvie, Torii Pharmaceutical, Maruho, Leo Pharma, Pfizer, Mitsubishi Tanabe, and Otsuka Pharmaceutical and is a member of an industry-academia collaboration course with Maruho. KT, KH, NK, KI, TT, and YY are employees of Maruho. KK has received consulting fees, honoraria, grant support, and/or lecture fees from AbbVie, Amgen, Eli Lilly, Kyowa Kirin, Japan Tobacco, LEO Pharma, Maruho, Mitsubishi Tanabe, Ono Pharmaceutical, Pfizer, Procter & Gamble, Sanofi, Regeneron, Taiho, and Torii Pharmaceutical. The remaining authors state no conflict of interest.
