# Corrigendum to ‘Plasma proteomic profiles of patients with atopic dermatitis with moderate-to-severe pruritus treated with a single dose of nemolizumab’ JID Innovations, Volume 6, Issue 3, May 2026, 100457

**DOI:** 10.1016/j.xjidi.2026.100469

**Published:** 2026-04-08

**Authors:** Saeko Nakajima, Hajime Iizuka, Kayo Taira, Kentaro Tanaka, Kei Hashimoto, Noriaki Kaneda, Kotaro Iwasaki, Takuya Takafuji, Yoshihito Yamada, Kenji Kabashima

**Affiliations:** 1 Department of Dermatology, Kyoto University Graduate School of Medicine, Kyoto, Japan; 2 Department of Drug Discovery for Inflammatory Skin Diseases, Kyoto University Graduate School of Medicine, Kyoto, Japan; 3 Hosui General Medical Clinic, Research Institute of Psoriasis, Sapporo, Japan; 4 SOUSEIKAI Hakata Clinic, Fukuoka, Japan; 5 Kyoto R&D Center, Maruho, Kyoto, Japan; 6 Medical Affairs Department, Maruho, Osaka, Japan; 7 A∗STAR Skin Research Labs (A∗SRL), Agency for Science, Technology and Research (A∗STAR), Singapore, Singapore; 8 Skin Research Institute of Singapore (SRIS), Agency for Science, Technology and Research (A∗STAR), Singapore, Singapore; 9 Singapore Immunology Network (SIgN), Agency for Science, Technology and Research (A∗STAR), Singapore, Singapore

The authors regret parts ‘e’ & ‘f’ are missing from published figure-4 in this article. Please see the corrected figure 4 below.

**Figure:**
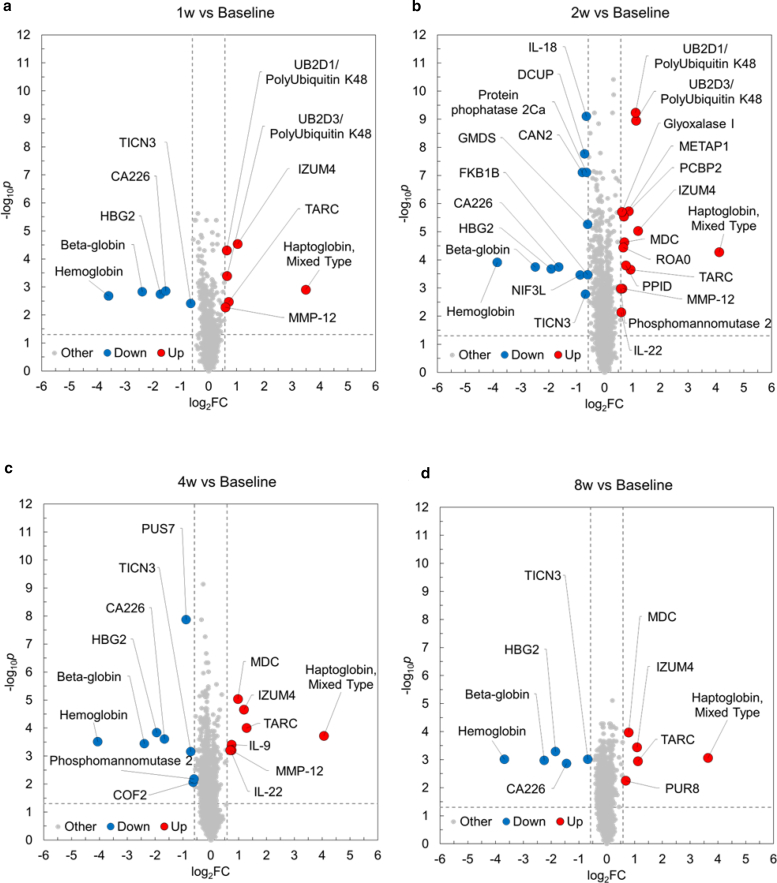

**Figure:**
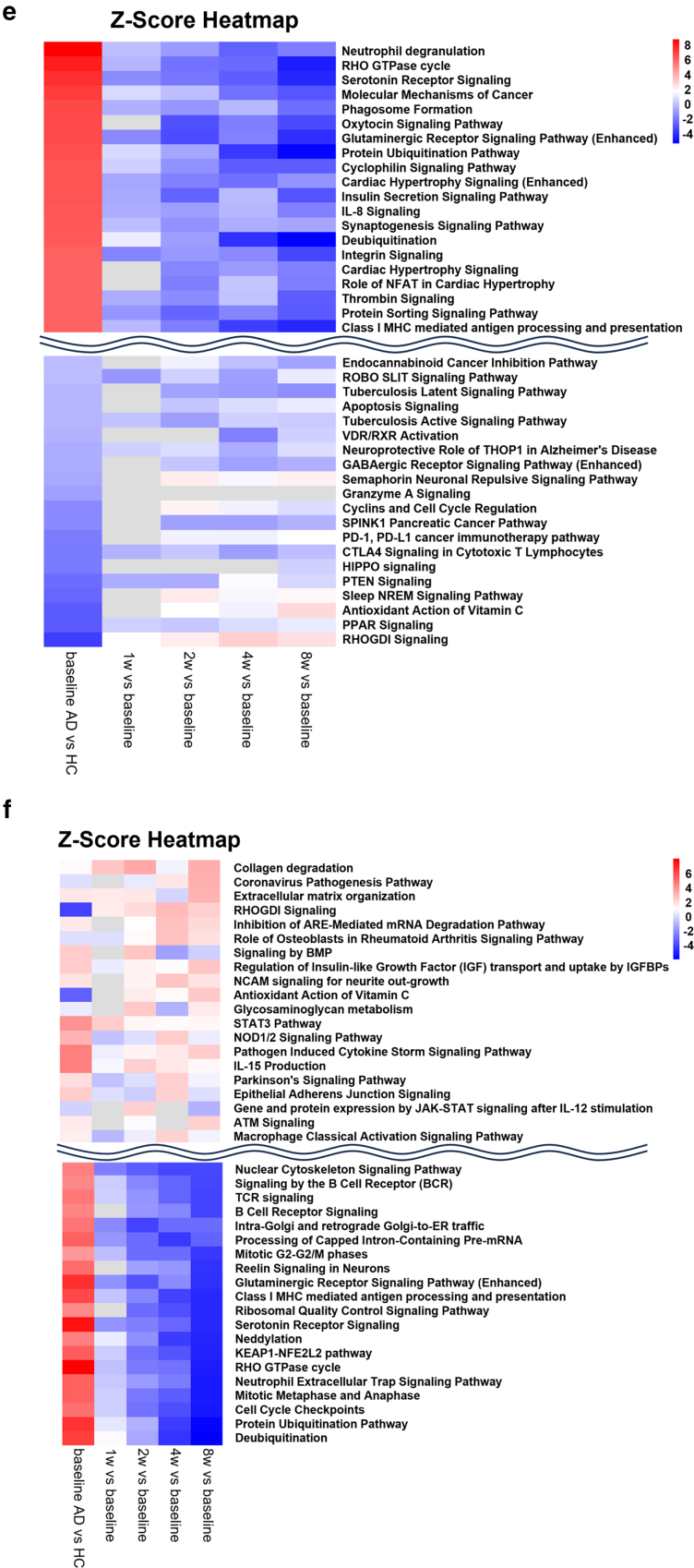

The authors would like to apologize for any inconvenience caused.

